# Dissecting the relationships of IgG subclasses and complements in membranous lupus nephritis and idiopathic membranous nephropathy

**DOI:** 10.1371/journal.pone.0174501

**Published:** 2017-03-23

**Authors:** Woong Na, Kijong Yi, Young Soo Song, Moon Hyang Park

**Affiliations:** 1 Department of Pathology, College of Medicine, Hanyang University, Seoul, Korea; 2 Department of Pathology, Konyang Univsersity Hospital, Daejeon, Korea; Instituto Nacional de Ciencias Medicas y Nutricion Salvador Zubiran, MEXICO

## Abstract

Membranous lupus nephritis (MLN) and idiopathic membranous nephropathy (IMN) are kidney diseases with similar morphology, but distinct etiologies, both producing glomeruli with immune deposits. Immunoglobulins and complements, the main components of the deposits, can be detected by immunofluorescence (IF) microscopy. Previous researches characterized the immune deposits only individually, but not the interactions between them. To study these relationships we analyzed an IF profile of IgG subclasses and complements (IgG1, IgG2, IgG3, IgG4, C3, C1q, and C4) in 53 and 95 cases of biopsy-confirmed MLNs and IMNs, respectively, mainly using information theory and Bayesian networks. We identified significant entropy differences between MLN and IMN for all markers except C3 and IgG1, but mutual information (a measure of mutual dependence) were not significantly different for all the pairs of markers. The entropy differences between MLN and IMN, therefore, were not attributable to the mutual information. These findings suggest that disease type directly and/or indirectly influences the glomerular deposits of most of IgG subclasses and complements, and that the interactions between any pair of the markers were similar between the two diseases. A Markov chain of IgG subclasses was derived from the mutual information about each pair of IgG subclass. Finally we developed an integrated disease model, consistent with the previous findings, describing the glomerular immune deposits of the IgG subclasses and complements based on a Bayesian network using the Markov chain of IgG subclasses as seed. The relationships between the markers were effectively explored by information theory and Bayesian network. Although deposits of IgG subclasses and complements depended on both disease type and the other markers, the interaction between the markers appears conserved, independent from the disease type. The disease model provided an integrated and intuitive representation of the relationships of the IgG subclasses and complements in MLN and IMN.

## Introduction

Membranous lupus nephritis (MLN) and idiopathic membranous nephropathy (IMN) are morphologically similar renal diseases exhibiting subepithelial immune deposits mainly composed of immune complexes and/or complements with minimal inflammatory reactions [1–5]. Though morphologically similar, the diseases are etiologically distinct and should be differentiated because of their clinical importance. MLN is one of the complications of systemic lupus erythematosus (SLE), whose main antigens include autoantigens against dsDNA, histones, and ribonucleoproteins [6]. In IMN, the M-type phospholipase A2 receptor is believed to be the target antigen [7,8]. The deposition of immune complexes against these antigens in the subepithelial portion of the glomerulus is a key pathologic feature of these diseases and the cause of the main clinical symptoms such as proteinuria and nephrotic syndrome.

Because IgG is a key component of these immune deposits, special attention has been given to them. According to the early results, IgG1, IgG2, and IgG3 tend to be highly expressed in MLN, and IgG1 and IgG4 in IMN [9–13]. Though a tight association between IgG subclasses and disease entities was revealed in these studies, only the behavior of individual markers was compared and the interactions between IgG subclasses in MLN and IMN were not considered. The glomerular deposits of a marker may be dependent not only on disease type, but also on the other markers.

We previously attempted to replicate the experimental findings and to develop more advanced data analysis procedures [12]. Specifically we used heatmap visualization with hierarchical clustering to reveal differential patterns of IgG subclass deposits between these diseases, and then developed predictive models using decision trees to estimate how much they improved diagnostic accuracy compared to naïve analysis. While these research goals were achieved, the interactions between IgG subclasses were still not clearly revealed.

According to another study performed by Huang et al., IgG1 was predominant in early IMN unlike late IMN where IgG4 was known to be predominant [14]. In data analysis, they introduced the concept of predominance and codominance to select the most representative markers. Although this study provided new insights on the role of IgG subclasses in IMN and more advanced data analysis procedures beyond individual marker analysis were developed, again the detailed interactions between IgG subclasses were not revealed.

Information theory provides a set of metrics useful in exploring the relationships between multiple random variables. These metrics can be directly applied to IgG subclasses to describe their behavior in MLN and IMN. Entropy (Shannon entropy) is a measure of the uncertainty of a random variable having a specific probability distribution [15], and mutual information is a measure of the mutual dependence of two random variables [16]. By measuring entropy and mutual information we can estimate how much information a variable contains and how much of it is shared with another variable. These properties of entropy and mutual information are very useful for global evaluation of the relationships between multiple biomarkers and constructing a more complete disease model.

Another approach to evaluating the relationships between random variables is to construct a Bayesian network. A Bayesian network is a graphical model that represents probabilistic relationships between random variables using a directed acyclic graph (DAG) [17]. The structure of the network can be obtained directly from data, but it is very sensitive to noise, thus requiring large amounts of data. Therefore it is desirable to identify the probable structure of a network before learning the structure from data, using prior knowledge or information obtained from other sources.

In this study, we investigated the relationships including interactions of IgG subclasses and/or complements using information theory approaches, and attempted to build integrated disease models for MLN and IMN using Bayesian networks. In addition to IgG subclasses, complements were analyzed together because complements are important immune mediators activated by immunoglobulins and easy to identify by IF microscopy. When developing the structure of the Bayesian network, selection criteria were applied using the information on IgG subclasses and complements in MLN and IMN obtained from information theory-based exploration of the data.

## Materials and methods

### 1. Case selection and data collection

In our department IF staining for IgG subclasses, C3, C1q, and C4 has been routinely conducted in cases of MLN and IMN. The present data were collected from the database of pathology reports from 2004 to 2015. The cases of MLN were limited to those where the histologic findings were compatible with class V lupus nephritis according to the International Society of Nephrology/Renal Pathology Society 2003 classification of LN [18] and antinuclear antibodies were detected at the time of onset or during the progress of the disease. The cases of IMN were limited to those where the histologic findings fitted the diagnostic criteria for IMN and no secondary etiologies were found at the onset or during the progression of the disease. Among 64 cases of MLN and 156 cases of IMN found by database query, we selected 148 cases, 53 of MLN and 95 of IMN based on the diagnostic criteria, sample quality, and availability of data. In the departmental protocol for renal biopsy, IF intensities for these markers are semiquantitatively evaluated in five renal compartments (glomerular capillary wall, mesangium, tubule, interstitium, and blood vessel) on a scale of 0 to 3. Only the data for the glomerular capillary wall and mesangium were used in our analysis because these data were considered most informative. To obtain more robust estimates of metrics of information theory and structures of Bayesian network, IF intensities were dichotomized into positive if raw IF intensities were 1, 2, or 3 and negative if 0. We, therefore, used either raw or dichotomized IF intensities depending on the type of analysis (PAC: raw data, analyses using information theory and Bayesian network: dichotomized data). To evaluate inter-observer variability, two pathologists (MP & WN) independently undertook IF scoring for 14 feasible cases and chance-corrected agreement (Cohen’s kappa) were 0.70 and 0.77 for raw and dichotomized IF intensities, respectively. This study was carried out in accordance with the code of ethics of the World Medical Association (Declaration of Helsinki), and was approved by the Institutional Review Board (IRB) of Hanyang University Hospital.

### 2. Analysis of global trends of IgG subclasses and complements in MLN and IMN

Before conducting the main analyses, we used principle component analysis (PCA) to identify global data trends because our data were of high dimensionality, and the pattern of high dimensional data is usually explored by PCA with visualization [19]. Specifically, data for 14 variables (7 IF markers x 2 compartments) were mapped into a space with new orthogonal axes. This space was constructed by linear transformation of each data point so as to explain the variability of the data in the most probable way. Using PCA and visualization, we attempted to see whether MLN and IMN were clearly distinct and whether there were multiple clusters indicating multiple disease subtypes.

### 3. Exploring the relationships between each pair of the IgG subclasses and complements in MLN and IMN using information theory

We explored the relationships between any biomarkers that could be represented as random variables in MLN and IMN using Shannon entropy and mutual information instead of Pearson’s correlation because every data point of our raw data was ordinal. The biomarkers included IF intensities of IgG1, IgG2, IgG3 IgG4, C3, C1q, and C4 and the disease type (whether a sample corresponded to MLN or IMN). By using entropy and mutual information, the dependence and independence of multiple random variables can be easily assessed.

**Fig 1:**
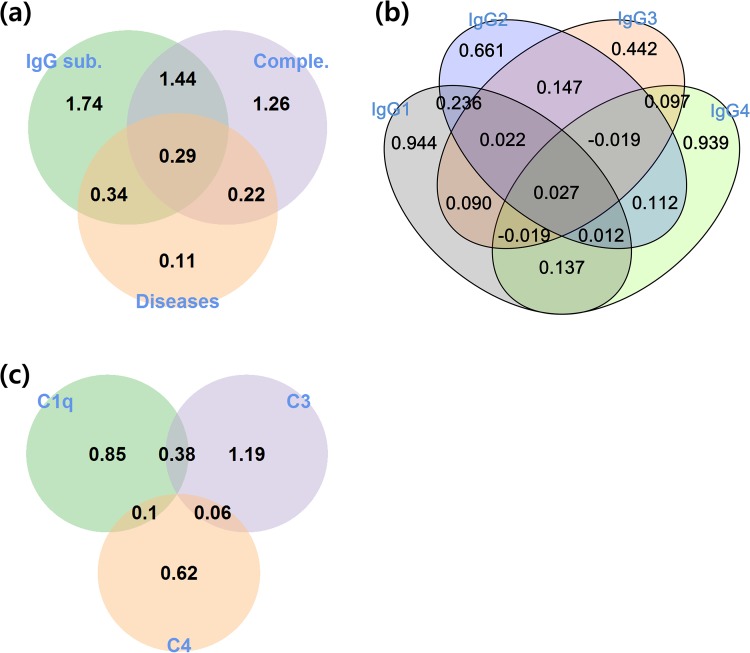
Information diagrams for IgG subclasses, complements, and disease type. Information diagram for IgG subclasses, complements, and disease type (a), for IgG subclasses (b), and for complements (c). Numbers in the diagram refer to bits.

As the first step in the analysis using information theory we constructed information diagrams for various queries concerning IgG subclasses, complements, and disease type (Fig 1). An information diagram is an integrated and intuitive representation of the relationships between multiple random variables in a Venn diagram-like structure which is suitable for visualizing entropy, mutual information, and other information. An information diagram can give the answers to various queries such as how much of a variable is uncertain, whether two variables are independent, and how much one can know the value of a given variable using the values of the other variables.

As a second step, we compared the differences of entropies and mutual information between MLN and IMN. By identifying significant differences of entropies between MLN and IMN, we can estimate which markers are differently expressed in MLN and IMN. Likewise if significant differences of mutual information between two markers are not readily shown, it may imply the conservation of the interaction between the two independent from the disease type. We used permutation tests to examine statistical significance, first obtaining the null distribution by permuting the disease state labels (MLN or IMN) and then computing the statistics.

### 4. Disease model construction based on a Bayesian network

To provide an integrated view of the relationships of IgG subclasses and complements in MLN and IMN, we constructed a disease model based on a Bayesian network, which is a graph model consisting of IgG subclasses, complements, and the disease type. The structure of a Bayesian network can be directly derived from the data, and this process is called structural learning. The results of entropy and mutual information analysis are used as prior knowledge during the structural learning. During this process important relationships are selected and less important ones are filtered. The Tabu learning algorithm was selected for the structural learning because it is fast and robust, and widely used [20]. For actual implementation, we used the R bnlearn package because the Tabu algorithm was already implemented in this package [21].

## Results

### 1. Distinct IgG subclass and complement profiles in MLN and IMN

**Fig 2:**
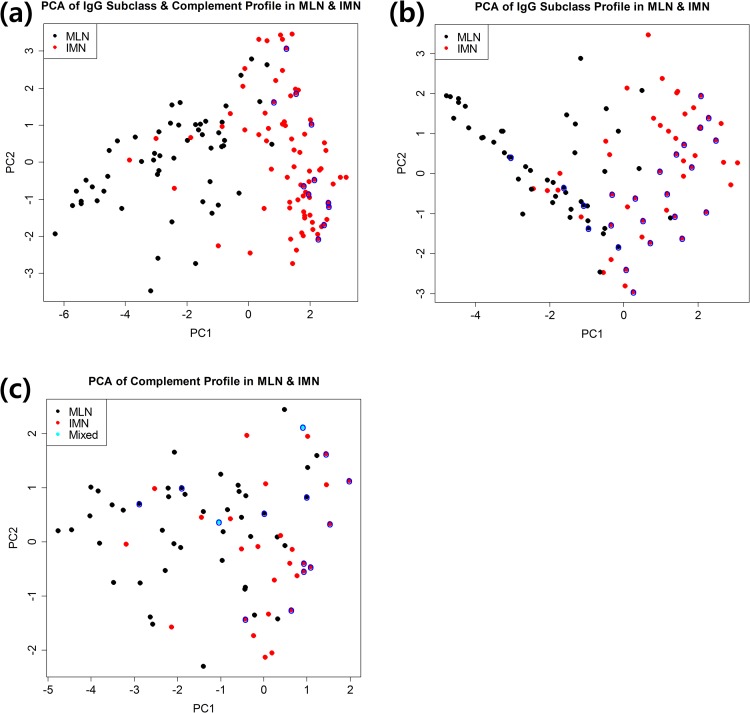
Global trends of the IgG subclasses and complements in MLN and IMN estimated by Principal Component Analysis (PCA). PCA of the IgG subclass and complements profile (a), profile of the IgG subclass only (b), and profile of complements only (c). Blue circles surrounding black, red, or cyan dots indicate that there is more than one case with the same profile. “Mixed” in (c) indicates that MLN and IMN cases have the same complement profile.

We investigated the global trends of IgG subclasses and complement profiles in MLN and IMN using principal component analysis (PCA) (Fig 2). MLN and IMN were differentially distributed in the PCA-transformed space although there was a small area of mixed MLN and IMN cases. This result implies that IgG subclasses and complement profiles are distinctly different in MLN and IMN (Fig 2(a)). We looked for possible subclusters, which suggest the presence of subtypes of a disease, but failed to identify any distinct subclusters in MLN or IMN.

We also conducted PCAs of IgG subclass profiles only, and complement profiles only (Fig 2(b) & 2(c), respectively). PCA of the IgG subclass profiles revealed a slightly less distinct distribution of MLN and IMN cases than the total profile. PCA of the complement profiles yielded rather haphazardly arranged data points in terms of disease state. These findings suggest that IgG subclass profiles and complement profiles provide complementary information about disease type.

Although differential distribution of MLN and IMN were identified by PCA, it was difficult to identify specific roles of a marker or a marker set only using PCA. Subsequently these data, therefore, were analyzed using information theory and Bayesian network.

### 2. Analysis using information theoretical measures has suggested that the interactions between any pair of IgG subclasses and/or complements may not be different in MLN and IMN

To more accurately define the features of IgG subclasses and complements in MLN and IMN, we computed various metrics of information theory that can be used to quantify unique and/or shared portions of information concerning multiple random variables. These quantities can yield insights into the relationships between IgG subclasses, complements, and disease type.

We constructed information diagrams for 1) IgG subclass, complement, and disease type, 2) IgG subclasses, and 3) complements (Fig 1). The Information diagram for IgG subclass, complement, and disease type showed that about 90% of disease type information was shared with either IgG subclasses or complements, and IgG subclasses and complements share > 45% of their own information (Fig 1(a)). The IgG subclasses shared more information with disease type than the complements did. These findings indicate that in most cases disease type could be inferred from the IgG subclasses and complements, and that the former are more informative about disease type than the latter. These findings could also be related to the results of PCAs. The information diagram for the IgG subclasses showed that IgG4 appeared to be relatively independent of the other three IgG subclasses, which is consistent with the known uniqueness of IgG4 in many diseases (Fig 1(b)) [22–26]. The information diagram for complements showed that C1q and C3 have more information than C4, and that C4 is relatively independent of C1q and C3.

**Fig 3:**
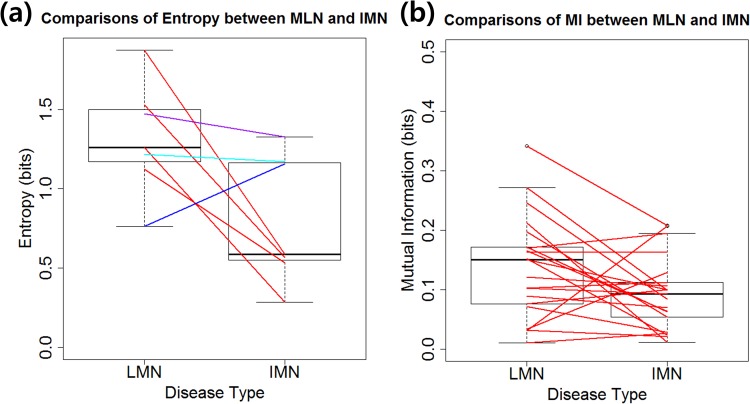
Comparison of entropy and Mutual Information (MI) in MLN and IMN. The differences of entropy between LMN and IMN for seven IgG subclass markers and complements (a), and differences of mutual information for 21 pairs of seven markers (b) are illustrated. The purple, cyan, and blue lines in (a) indicates the difference of entropy for C3, IgG1, and IgG4, respectively.

To identify the differences of behavior of IgG subclasses, complements, and their interactions in MLN and IMN, we investigated how entropy and mutual information changed depending on the disease type. All the entropies of the IgG subclasses or complements except IgG4 decreased when one changed from LMN to IMN (Fig 3(a)) while mutual information only changed minimally (Fig 3(b)). We investigated whether these changes were statistically significant by permutation testing. Interestingly all the mutual information between any pair of IgG subclasses or complements did not differ significantly between MLN and IMN (S1 Table) whereas all the entropies of the IgG subclasses and complements were significantly different except IgG1 and C3 (Table 1). To confirm that the similar values of mutual information in MLN and IMN were due to similar probability distributions, we compared the joint probability distributions of each pair of the markers in MLN with those in IMN by visual inspection. All the joint distributions appeared similar. Differential expression of most of IgG subclasses and complements in MLN vs. IMN is well known, but the interactions between these markers may not differ in the two diseases. It should be noted that the entropy differences between MLN and IMN appeared not attributable to the mutual information. These findings suggest that the disease type influences the glomerular deposition of most of the markers directly and/or indirectly via the other markers, but that their interactions are similar in the two disease conditions, suggesting that IgG subclasses, complements, and each interactions between them would be elements of a subsystem of an immune systems in MLN and IMN. These findings prompted us to construct a single disease model applicable to both MLN and IMN using IgG subclasses and complements, rather than separate disease models for MLN and IMN.

**Table 1:** Comparisons of entropy in MLN and IMN.

Marker	Entropy in MLN	Entropy in IMN	p value
IgG1	1.22	1.17	0.75
IgG2	1.53	0.57	< 0.0001
IgG3	1.26	0.29	< 0.0001
IgG4	0.76	1.16	0.0012
C1q	1.87	0.59	0.31
C3	1.47	1.32	0.02
C4	1.12	0.54	< 0.0001

**Fig 4:**
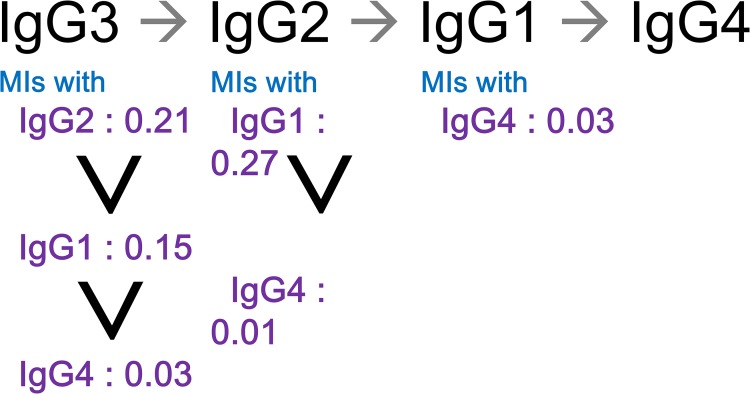
Markov chain and Mutual Information (MI) of the IgG subclasses. The structure of the Markov chain was inferred from the MIs, on the assumption that IgG1, IgG2, IgG3, and IgG4 form a single chain Markov chain, The MIs are represented in bits.

The mutual information between IgG1, IgG2, IgG3, and IgG4 enabled us to construct a Markov chain, IgG3→IgG2→IgG1→IgG4 (Fig 4). According to data-processing inequality, if there is a Markov chain, X→Y→Z, then I(X;Y)≥I(X;Z) [27]. If one assumes that X, Y, Z form a single chain Markov chain in all possible ways, the reverse is also true. Based on the assumption that IgG1, IgG2, IgG3, IgG4 form a single chain Markov chain, the only structure satisfying the conditions for the mutual information was IgG3→IgG2→IgG1→IgG4. This assumption was justifiable in the initial stage of model construction, since selecting a simple structure is the better option, and it can be changed to a more complex one if severe bias is found.

### 3. Integrated disease model of MLN and IMN representing the interactions among IgG subclasses and complements

**Fig 5:**
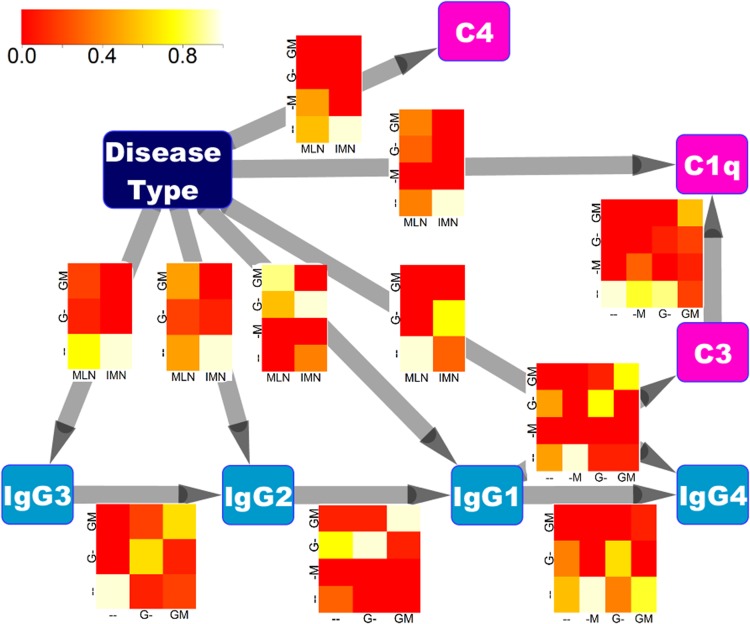
A model of IgG subclass and complement deposition in the glomeruli in MLN and IMN. The deposits of the IgG subclasses and complements in MLN and IMN are illustrated as a Bayesian network. Nodes consist of disease type (MLN or IMN), IgG subclass, and complement, and edges with arrows represent cause and effect relationships. Conditional probabilities of the target variable given the source variable in each edge are represented as heatmaps over edges. In each heatmap, the x axis and y axis represent source and target variable, respectively, and each conditional probability is color coded. The values of glomerular deposits of IgG subclasses and complements are represented as ‘—‘,’-M’, ‘-G’, ‘GM’ each indicating no deposits (‘—‘), deposit only in the mesangium (‘-M’), deposit along the glomerular capillary wall (‘G-’), and deposit both in the mesangium and along the glomerular capillary wall (‘GM’), respectively. A column or row of the heatmap is omitted for simplicity if all the values in the corresponding line are 0.

To concisely represent glomerular deposits of IgG subclasses and complements in MLN and IMN, we developed a disease model based on a Bayesian network (Fig 5). The results of the analyses using information theory guided the structure of the network. First the robustness of the interactions between any pair of markers when switching from MLN to IMN suggested an integrated model rather than separate models for MLN and IMN. Second the inferred Markov chain (IgG3→IgG2→IgG1→IgG4) was used as a seed in constructing the network.

The network consisted of 8 nodes representing IgG subclasses, complements, and disease type (MLN or IMN), and 11 directed edges representing cause and effect relationships between the nodes, with five edges between the markers and six nodes between disease type and markers (Fig 5). A part of the network contained the Markov chain (IgG3→IgG2→IgG1→IgG4) used as a seed. All the markers were directly influenced by the disease type except C3 whose entropy was not significantly different in MLN and IMN. The glomerular deposits of IgG1, IgG2, IgG4, and C1q were determined by two factors, the disease type and the value of the preceding marker. C4 did not have any relation with the other markers and was only influenced by the disease type. Interestingly there were no edges from complements to IgG subclasses, and IgG1 directly influenced C3. These findings partly justify the model because complements are directly activated by immunoglobulins and we could not find any reports that complements directly activate immunoglobulins.

The network also illustrated the strengths of the cause and effect relationships in terms of conditional probabilities. We could easily confirm from the heatmaps that IgG2, IgG3, and C4 are rarely expressed in IMN and IgG4 in MLN (Fig 5). Our model thus describes all the important relationships between the markers effectively in one diagram.

## Discussion

The core components of both MLN and IMN are subepithelial immune deposits, but the chemical composition of the deposits tends to be different [9–11]. However because the composition of the immune deposits in MLN and IMN was diverse and complex, previous researches focusing on the behavior of individual markers was insufficient to model the global nature of the deposits. As we believed that the interactions between the markers would have a considerable effect on the IgG subclasses and complement profiles in MLN and IMN, we focused on quantifying them. We proved that the interactions were similar in MLN and IMN. These findings are significant by itself and also provided an important basis for an integrated disease model. The integrated disease model was consistent with the previously known behavior of IgG subclasses and complements in MLN and IMN, and provided a concise description of the IgG subclasses and complement profiles in MLN and IMN.

Information theory made key contributions to quantifying the interactions between the markers in this research. Although Pearson’s correlation is the metric of choice in evaluating the interactions between two random variables in most nephrology research, it is insensitive to non-continuous variables, only captures positive or negative relationships, and is sensitive to noise in the data [28]. Because the variables representing glomerular immune deposits were ordinal, mutual information was a far better choice than Pearson’s correlation. Mutual information is easily integrated with the other metrics of information theory and all the metrics of information theory can be represented as an information diagram providing clues about relationships, including the dependencies and independencies among multiple variables. It was particularly useful when testing whether a similar kind of interaction between markers exists in MLN and IMN, because the interactions can be assessed from the joint distributions, and mutual information is a quantity totally dependent on the joint distributions. The results of analyses using the metrics of information theory were significant by themselves and also guided the direction of subsequent analyses, particularly of the structure of the Bayesian network. These kinds of analytic schemes are applicable in other research if multiple random variables are thought to interact with each other, and most of the random variables are categorical or ordinal.

Using a Bayesian network, we were able to concisely represent the complex behaviors of the IgG subclasses and complements in MLN and IMN so providing an integrated knowledge resource. The resulting model displayed all the important information regarding IgG subclasses, complements, MLN, and IMN in one diagram, and was consistent with the previous findings about the markers [9–14]. The existence of an edge starting from IgG and ending in the complements, and the absence of edges starting from complement and ending with IgG may also validate the model because immunoglobulins activate complements by physical contact, and not vice versa [29,30]. It was also easily visualized from the heatmaps that IgG2, IgG3, and C4 are rarely expressed in IMN and IgG4 in MLN. These findings may suggest that the disease model can be served as an alternative representation of previously known findings and new hypotheses can be generated from this model.

One thing to be cautious about when interpreting the Bayesian network is that the edges of the network do not indicate the existence of physical interaction between connected nodes but rather a flow of information. For example, it would not be reasonable to assume that IgG3 physically activates IgG2; instead the molecules responsible for activating IgG2 would be other immune mediators such as cytokines. This network, therefore, could be extended by incorporating experimental data on such immune mediators without greatly changing the structure of the network. It is also worth noting that the current network is the most informative one available until such additional data have been acquired.

The disease model represented as a Bayesian network can be also used in clinical decision making where differentiation between MLN and IMN is required based on IgG subclass and complement profile. When the diagnosis is ambiguous and IF intensities of IgG subclasses and complements are given, we can obtain conditional probabilities of each of MLN and IMN using the inference rule of the network, and the one with higher conditional probability would be chosen as the most probable diagnosis. The diagnostic accuracy of the network can be compared with a similar predictive model we constructed using decision tree [12]. The inference of the diagnosis is also feasible even when IgG subclass or complement profile has some missing values. Likewise the disease model is useful in many different ways such as representing the complex relationships and inferring the diagnosis, not confined to our description.

Although the interactions between the markers in MLN and IMN were quantified using mutual information, the detailed mechanisms of the interactions cannot be specified by mutual information alone. For example, one cannot conclude whether IgG3 activates or inactivates IgG2 using only mutual information. Further studies are needed to specify such mechanisms. However the model would still be useful for designing experiments to specify these mechanisms because less important interactions have presumably already been filtered out. Without such guidance, the experimental cost would be too high. It would be also useful in exploring the behaviors of immune mediators other than IgG subclasses or complements. Let us suppose that substance A and B are highly secreted dominant immune mediators in MLN and IMN, respectively. If the experimental results were that A activated both IgG2 and IgG3 the behaviors of substance B would be quite limited because the interactions between any pair of IgG subclasses and complements should be similar in MLN and IMN. These things would be helpful in designing the experiments about the substance B.

The kinetics of immune deposits in the glomeruli in MLN and IMN are also important for understanding the roles of IgG subclasses and complements in these diseases. In particular, IgG subclass switching is a crucial event determining the composition of the immune deposits [14]. The identity of immune mediators and transcription factors involved in class switching would partly account for the particular profiles of the immune deposits in MLN and IMN [31–34]. Furthermore temporal associations between these factors need to be considered to formulate a complete model. In connection with class switching, it is interesting that the Markov chain, IgG3→IgG2→IgG1→IgG4 generated using mutual information has a similar sequence as that of the IgG subclass genes in the genome, IgG3→IgG1→IgG2→IgG4 [35]. This suggests that the immune deposits evaluated at the time of diagnosis might be footprints of multiple rounds of subclass switching, an idea that can be tested by simulation studies. Additional simulation studies could generate the most likely time-dependent subclass switching patterns fitting the IgG subclass and complement profiles.

One of limitations of our study is that the raw data of our study was obtained from the pathology report without reviewing the whole cases. However these kinds of study using electrical health records for medical research are increasing in number [36]. If adequate quality control procedures have been regularly conducted, much of biases would not be found compared to traditional well-controlled studies. In future studies, we could explore these issues by investigating the consistency of the disease model after adding random noises.

In conclusion, we have proved using information theory the similarity of the interactions between any pair of markers of the IgG subclasses and complements when switching from MLN to IMN, and have developed a disease model of MLN and IMN dealing with the glomerular deposits of IgG subclasses and complements. It should be also noted that our disease model effectively visualized many of known or latent findings of the IgG subclasses and complements in MLN and IMN in one diagram. It would be interesting to investigate whether our model is valid only for MLN and IMN or also for other glomerular diseases. Although further studies are necessary to augment this model by incorporating immune mediators, class switching events, and temporal factors, it provides a foundation for constructing more advanced ones.

## Supporting information

S1 Table Comparisons of mutual information in MLN and IMN. (DOCX)

## References

[1] Donadio JV Jr., Burgess JH, Holley KE. Membranous lupus nephropathy: a clinicopathologic study. Medicine. 1977;56(6):527–36. 916891 10.1097/00005792-197711000-00007

[2] Donadio JV Jr., Torres VE, Velosa JA, Wagoner RD, Holley KE, Okamura M, et al Idiopathic membranous nephropathy: the natural history of untreated patients. Kidney international. 1988;33(3):708–15. 3367560 10.1038/ki.1988.56

[3] Franklin WA, Jennings RB, Earle DP. Membranous glomerulonephritis: long-term serial observations on clinical course and morphology. Kidney international. 1973;4(1):36–56. 4723993 10.1038/ki.1973.78

[4] Friend PS, Kim Y, Michael AF, Donadio JV. Pathogenesis of membranous nephropathy in systemic lupus erythematosus: possible role of nonprecipitating DNA antibody. British medical journal. 1977;1(6052):25 831973 10.1136/bmj.1.6052.25 PMC1603593

[5] Jennette JC, Iskandar SS, Dalldorf FG. Pathologic differentiation between lupus and nonlupus membranous glomerulopathy. Kidney international. 1983;24(3):377–85. 6358633 10.1038/ki.1983.170

[6] Riemekasten G, Hahn BH. Key autoantigens in SLE. Rheumatology. 2005;44(8):975–82. 10.1093/rheumatology/keh688 15901907

[7] Beck LH Jr. Membranous nephropathy and malignancy. Seminars in nephrology. 2010;30(6):635–44. 10.1016/j.semnephrol.2010.09.011 21146128

[8] Beck LH Jr., Bonegio RG, Lambeau G, Beck DM, Powell DW, Cummins TD, et al M-type phospholipase A2 receptor as target antigen in idiopathic membranous nephropathy. The New England journal of medicine. 2009;361(1):11–21. 10.1056/NEJMoa0810457 19571279 PMC2762083

[9] Imai H, Hamai K, Komatsuda A, Ohtani H, Miura AB. IgG subclasses in patients with membranoproliferative glomerulonephritis, membranous nephropathy, and lupus nephritis. Kidney international. 1997;51(1):270–6. 8995742 10.1038/ki.1997.32

[10] Kuroki A, Shibata T, Honda H, Totsuka D, Kobayashi K, Sugisaki T. Glomerular and serum IgG subclasses in diffuse proliferative lupus nephritis, membranous lupus nephritis, and idiopathic membranous nephropathy. Internal medicine. 2002;41(11):936–42. 12487163 10.2169/internalmedicine.41.936

[11] Ohtani H, Wakui H, Komatsuda A, Okuyama S, Masai R, Maki N, et al Distribution of glomerular IgG subclass deposits in malignancy-associated membranous nephropathy. Nephrology, dialysis, transplantation: official publication of the European Dialysis and Transplant Association—European Renal Association. 2004;19(3):574–9. 10.1093/ndt/gfg616 14767011

[12] Song YS, Min KW, Kim JH, Kim GH, Park MH. Differential diagnosis of lupus and primary membranous nephropathies by IgG subclass analysis. Clinical journal of the American Society of Nephrology: CJASN. 2012;7(12):1947–55. 10.2215/CJN.04800511 23024158 PMC3513749

[13] Zuniga R, Markowitz GS, Arkachaisri T, Imperatore EA, D'Agati VD, Salmon JE. Identification of IgG subclasses and C-reactive protein in lupus nephritis: the relationship between the composition of immune deposits and FCgamma receptor type IIA alleles. Arthritis and rheumatism. 2003;48(2):460–70. 10.1002/art.10930 12571856

[14] Huang CC, Lehman A, Albawardi A, Satoskar A, Brodsky S, Nadasdy G, et al IgG subclass staining in renal biopsies with membranous glomerulonephritis indicates subclass switch during disease progression. Modern pathology: an official journal of the United States and Canadian Academy of Pathology, Inc. 2013;26(6):799–805. 10.1038/modpathol.2012.237 23328976

[15] Stewart JJ, Lee CY, Ibrahim S, Watts P, Shlomchik M, Weigert M, et al A Shannon entropy analysis of immunoglobulin and T cell receptor. Molecular immunology. 1997;34(15):1067–82. 9519765 10.1016/s0161-5890(97)00130-2

[16] Shlush LI, Bercovici S, Wasser WG, Yudkovsky G, Templeton A, Geiger D, et al Admixture mapping of end stage kidney disease genetic susceptibility using estimated mutual information ancestry informative markers. BMC medical genomics. 2010;3:47 10.1186/1755-8794-3-47 20955568 PMC2975638

[17] Sesen MB, Nicholson AE, Banares-Alcantara R, Kadir T, Brady M. Bayesian networks for clinical decision support in lung cancer care. PloS one. 2013;8(12):e82349 10.1371/journal.pone.0082349 24324773 PMC3855802

[18] Weening JJ, D'Agati VD, Schwartz MM, Seshan SV, Alpers CE, Appel GB, et al The classification of glomerulonephritis in systemic lupus erythematosus revisited. Journal of the American Society of Nephrology: JASN. 2004;15(2):241–50. 14747370 10.1097/01.asn.0000108969.21691.5d

[19] Ringner M. What is principal component analysis? Nature biotechnology. 2008;26(3):303–4. 10.1038/nbt0308-303 18327243

[20] Duering M, Gonik M, Malik R, Zieren N, Reyes S, Jouvent E, et al Identification of a strategic brain network underlying processing speed deficits in vascular cognitive impairment. NeuroImage. 2013;66:177–83. 10.1016/j.neuroimage.2012.10.084 23153965

[21] Scutari M. Learning Bayesian networks with the bnlearn R package. arXiv preprint arXiv:09083817. 2009.

[22] Umemura T, Zen Y, Hamano H, Ichijo T, Kawa S, Nakanuma Y, et al IgG4 associated autoimmune hepatitis: a differential diagnosis for classical autoimmune hepatitis. Gut. 2007;56(10):1471–2. 10.1136/gut.2007.122283 17504944 PMC2000273

[23] Deshpande V. IgG4-Related Disease of the Gastrointestinal Tract: A 21st Century Chameleon. Archives of pathology & laboratory medicine. 2015;139(6):742–9. 26030243 10.5858/arpa.2014-0181-RA

[24] Sato Y, Notohara K, Kojima M, Takata K, Masaki Y, Yoshino T. IgG4-related disease: historical overview and pathology of hematological disorders. Pathology international. 2010;60(4):247–58. 10.1111/j.1440-1827.2010.02524.x 20403026

[25] Culver EL, Chapman RW. IgG4-related hepatobiliary disease: an overview. Nature reviews Gastroenterology & hepatology. 2016;13(10):601–12. 27625195 10.1038/nrgastro.2016.132

[26] Inoue D, Zen Y, Abo H, Gabata T, Demachi H, Kobayashi T, et al Immunoglobulin G4-related lung disease: CT findings with pathologic correlations. Radiology. 2009;251(1):260–70. 10.1148/radiol.2511080965 19221056

[27] Kinney JB, Atwal GS. Equitability, mutual information, and the maximal information coefficient. Proceedings of the National Academy of Sciences of the United States of America. 2014;111(9):3354–9. 10.1073/pnas.1309933111 24550517 PMC3948249

[28] Jeong HH, Leem S, Wee K, Sohn KA. Integrative network analysis for survival-associated gene-gene interactions across multiple genomic profiles in ovarian cancer. Journal of ovarian research. 2015;8:42 10.1186/s13048-015-0171-1 26138921 PMC4491426

[29] Heyman B. Regulation of antibody responses via antibodies, complement, and Fc receptors. Annual review of immunology. 2000;18:709–37. 10.1146/annurev.immunol.18.1.709 10837073

[30] Wernersson S, Kleinau S, Heyman B. Immune complex-mediated enhancement of antibody responses without induction of delayed-type hypersensitivity. Scandinavian journal of immunology. 2000;52(6):563–9. 11119261 10.1046/j.1365-3083.2000.00813.x

[31] Cerutti A. The regulation of IgA class switching. Nature reviews Immunology. 2008;8(6):421–34. 10.1038/nri2322 18483500 PMC3062538

[32] Kometani K, Nakagawa R, Shinnakasu R, Kaji T, Rybouchkin A, Moriyama S, et al Repression of the transcription factor Bach2 contributes to predisposition of IgG1 memory B cells toward plasma cell differentiation. Immunity. 2013;39(1):136–47. 10.1016/j.immuni.2013.06.011 23850379

[33] Peng SL, Szabo SJ, Glimcher LH. T-bet regulates IgG class switching and pathogenic autoantibody production. Proceedings of the National Academy of Sciences of the United States of America. 2002;99(8):5545–50. 10.1073/pnas.082114899 11960012 PMC122806

[34] Roper RL, Graf B, Phipps RP. Prostaglandin E2 and cAMP promote B lymphocyte class switching to IgG1. Immunology letters. 2002;84(3):191–8. 12413736 10.1016/s0165-2478(02)00185-2

[35] Collins AM, Jackson KJ. A Temporal Model of Human IgE and IgG Antibody Function. Frontiers in immunology. 2013;4:235 10.3389/fimmu.2013.00235 23950757 PMC3738878

[36] De Moor G, Sundgren M, Kalra D, Schmidt A, Dugas M, Claerhout B, et al Using electronic health records for clinical research: the case of the EHR4CR project. Journal of biomedical informatics. 2015;53:162–73. 10.1016/j.jbi.2014.10.006 25463966

