# Supplementary material for: Dissecting the relationships of IgG subclasses and complements in membranous lupus nephritis and idiopathic membranous nephropathy
Source: PLoS One. 2017 Mar 23;12(3):e0174501. doi: 10.1371/journal.pone.0174501 (PMC5363951; doi:10.1371/journal.pone.0174501)
Supplement: S1 Table — (DOCX) [file pone.0174501.s001.docx]

**S1 Table . Comparisons of mutual information (MI) in MLN and IMN**

| Markers | MI in MLN | MI in IMN | p value |
| --- | --- | --- | --- |
| IgG1, IgG2 | 0.27 | 0.10 | 0.24 |
| IgG1, IgG3 | 0.15 | 0.02 | 0.16 |
| IgG1, IgG4 | 0.03 | 0.13 | 0.40 |
| IgG1, C3 | 0.17 | 0.19 | 0.88 |
| IgG1, C4 | 0.10 | 0.11 | 0.87 |
| IgG1, C1q | 0.16 | 0.16 | 0.99 |
| IgG2, IgG3 | 0.21 | 0.01 | 0.09 |
| IgG2, IgG4 | 0.01 | 0.03 | 0.87 |
| IgG2, C3 | 0.17 | 0.06 | 0.46 |
| IgG2, C4 | 0.17 | 0.06 | 0.14 |
| IgG2, C1q | 0.25 | 0.08 | 0.33 |
| IgG3, IgG4 | 0.03 | 0.02 | 0.87 |
| IgG3, C3 | 0.07 | 0.03 | 0.58 |
| IgG3, C4 | 0.20 | 0.05 | 0.09 |
| IgG3, C1q | 0.09 | 0.07 | 0.85 |
| IgG4, C3 | 0.03 | 0.21 | 0.18 |
| IgG4, C4 | 0.10 | 0.09 | 0.90 |
| IgG4, C1q | 0.15 | 0.10 | 0.66 |
| C3, C4 | 0.08 | 0.10 | 0.71 |
| C3, C1q | 0.34 | 0.20 | 0.40 |
| C4, C1q | 0.12 | 0.11 | 0.88 |
